# O.6.3-6 The effects of an active breaks intervention on physical and cognitive performance: results from the I-MOVE study

**DOI:** 10.1093/eurpub/ckad133.286

**Published:** 2023-09-11

**Authors:** Alice Masini, Sofia Marini, Andrea Ceciliani, Giuseppe Barone, Marcello Lanari, Davide Gori, Laura Bragonzoni, Stefania Toselli, Rita Stagni, Mariacristina Bisi, Alessandra Sansavini, Alessia Tessari, Laura Dallolio

**Affiliations:** Department of Biomedical and Neuromotor Sciences, University of Bologna, Italy; Department for Life Quality Studies, University of Bologna, Italy; Department for Life Quality Studies, University of Bologna, Italy; Department for Life Quality Studies, University of Bologna, Italy; Pediatric Emergency Unit, S. Orsola University Hospital, Scientific Institute for Research and Healthcare (IRCCS) Azienda Ospedaliero-Universitaria di Bologna; Department of Biomedical and Neuromotor Sciences, University of Bologna, Italy; Department for Life Quality Studies, University of Bologna, Italy; Department for Life Quality Studies, University of Bologna, Italy; Department of Electrical, Electronic, and Information Engineering “Guglielmo Marconi”, University of Bologna, Italy; Department of Electrical, Electronic, and Information Engineering “Guglielmo Marconi”, University of Bologna, Italy; Department of Psychology “Renzo Canestrari”, University of Bologna, Italy; alice.masini7@unibo.it; Department of Psychology “Renzo Canestrari”, University of Bologna, Italy; alice.masini7@unibo.it; Department of Biomedical and Neuromotor Sciences, University of Bologna, Italy

## Abstract

**Purpose:**

The present quasi-experimental study aimed to evaluate the effects of active breaks intervention (ABs) to promote physical and cognitive improvement in primary school.

**Methods:**

The active breaks group (ABsG) performed 10 minutes of ABs three times per school day and the control group (CG) did normal lessons. The baseline and follow-up evaluation was conducted respectively in October-2019 and in May-2021. Cognitive performance was assessed using working memory test, physical performance was analysed with ActiGraph accelerometers and physical fitness tests, quality of life was monitored using the Paediatric Quality of Life questionnaire (PedsQL) and classroom behaviour was collected with an ad hoc questionnaire.

**Results:**

We enrolled 153 children (age:7.61±1.41, 54.2% males). Working memory significantly increased in the ABsG (ΔWM:1.30±1.17) than in CG (ΔWM:0.96±1.20). The 6-minutesCooper test increased in the ABsG (Δ:1.77±136.03) but not in CG (Δ:-156.42±187.53) p < 0.05. The weekly PA levels increased in both groups however the sedentary behaviour significantly increased both in ABsG and CG. Children reported improvements in their quality of school life including feeling better in class and in school when using ABs, moreover children improved their time on task behaviours in ABsG.

**Conclusion:**

The present study proven to be effective on children's physical and cognitive performance.

